# Advancing preclinical cancer models to assess clinically relevant outcomes

**DOI:** 10.1186/s12885-023-10715-7

**Published:** 2023-03-10

**Authors:** Anna Golebiewska, Ryan C. Fields

**Affiliations:** 1 NORLUX Neuro-Oncology Laboratory, Department of Cancer Research, Luxembourg Institute of Health, L-1210 Luxembourg, Luxembourg; 2 The Alvin J. Siteman Comprehensive Cancer Ceter, Department of Surgery, Washington University School of Medicine in St. Louis, St. Louis, USA

## Abstract

Cancer models are indispensable research tools for elucidating the mechanisms involved in tumor onset, progression and treatment resistance. They are key in evaluating therapeutics prior clinical trials. In this editorial, we invite contributions for a BMC Cancer’s Collection of articles addressing ‘*Advances in pre-clinical cancer models’* towards relivable outcomes at the preclinical stage.

Cancers, in particular those of the aggressive nature, form very dynamic ‘aberrant organs’ that ultimately profit from their hosts to develop and survive. Although certain histopathological and genetic traits are common among cancers of the same (sub-)type, in essence each patient tumor is unique. The inherent inter-patient and intra-tumoral heterogeneity present at the cellular and molecular levels is a major hurdle for experimental cancer modeling. It is becoming clear that preclinical models recapitulating the dynamic tumor ecosystem, composed of tumor cells embedded in the adequate tumor microenvironment (TME), will be key for improving success rates of therapeutics in the clinic. Still, the research community will have to accept the intrinsic limitations of preclinical cancer modeling and develop a battery of protocols aiming at answering diverse questions step-by-step with models tailored best to each individual biological hypothesis.

Preclinical cancer modeling came a long way since its infancy days as adherent 2D in vitro cell lines. Individual and collaborative international efforts over the years led to a large portfolio of in vitro cell lines derived from patient tumors, including thorough characterization at the genetic and pharmacological levels [1]. Although very useful for mechanistic studies, such cell lines undergo selection and adaptation in culture, due to e.g. lack of physical and biological pressure from the TME and non-physiological media composition. Together with the ongoing genetic drift and cell line misclassifications [2], these parameters ultimately lead to inadequate responses to therapeutics [3]. Numerous adaptations have emerged in recent years to improve in vitro conditions towards the 3D architecture and oxygen gradient. These include 3D growth as spheres, serum-free and physiological media, adapted oxygen levels as well as physical forces added by e.g. hanging drop cultures and bioreactor-based culture rotations [4]. A significant step forward came with the rise of tumor organoids. Although definition of the term ‘’organoid’’ is not clearly established, such cultures should represent a more complex organization than 3D spheres, allowing for a better preservation of the genetic and phenotypic heterogeneity. Importantly, organoids can be derived from less aggressive tumors, leading to an expanded diversity of patient-derived models [5, 6]. The ongoing efforts in developing cell printing [7] and tumor-on-chip technologies [8], incorporating diverse cell scaffolds, extracellular matrix components and microfluidics towards physiological gradients of soluble factors and oxygen, will lead to improved phenotypes of tumor cells ex vivo.

In vivo xenotransplantation of small tissue fragments in the flanks of rodents, i.e. patient-derived xenografts (PDXs), has been long considered as the most reliable option for the serial propagation of human tumors in vivo without a culture step [9, 10]. Similarly to tumor organoids, PDXs can be derived from more genetically diverse and less aggressive tumors [11]. Orthotopic implantations (i.e. PDOXs) are currently more favorable in the research community due to the more suitable TME in the organ of origin. However, due to technical drawbacks, the majority of orthotopic models are nowadays based on the implantation of enzymatically dissociated cells or primary organoids cultured short-term ex vivo [6]. If all steps of metastasis are essential, the implantation should be performed via blood or to the initial pre-metastatic organ, allowing for tumor cells to undergo a full process of cell migration from the primary site to distant organs. Importantly, most likely, these models will only be available in expert laboratories due to their high cost and advanced expertise.

The ongoing challenge in the field is the robust incorporation of the adequate cellular components of the TME to current models. Any in vitro cultures, including organoids, lead to an inevitable loss of non-neoplastic cells upon passaging. Upon xenografting, TME cells are immediately replaced by the host counterparts. The obligatory use of immunodeficient animals for xenografting leads to lack of the adaptive immune system. The long-standing solutions for investigating the TME are animal models, including genetically-engineered mouse models (GEMMs), chemically induced models and allogenic transplantations of cultured tumor cells of animal origin. The application of embryonic stem cell-derived chimeras and the development of CRISPR-based gene editing technologies have recently boosted the field. Despite being widely used for testing novel immunotherapeutics at the preclinical stage, these models are currently highly criticized for the lack of resemblance to human disease. The breaking step would be the incorporation of not only gene mutations, but also genetic chromosomal copy-number alterations. Another promising option is the inclusion of missing TME components back to the preclinical models based on the patient tumor material. Numerous co-culture protocols are emerging allowing to integrate different types of non-neoplastic cells into tumor cultures or to integrate tumor cells into normal organoids [12, 13]. While technological advances allow for cells printing on sophisticated scaffolds and the introduction of immune cells via tubes resembling blood vessels, the ongoing challenge lies in the continuous source of relevant non-neoplastic cells and in finding a medium composition fitting all cell types in the co-culture system. In vivo, humanized mice bring an additional promise: while the PBMC-based models allow for the incorporation of mature allogenic or autologous tumor cells, humanized models based on the CD34 + hematopoietic stem cells allow a wider experimental window and the incorporation of immune cells from the early onset of the tumor growth [9]. This includes newer humanized PDX models that are able to recapitulate the human immune system beyond T-cells, including components of the adaptive and innate immune system [14]. These sophisticated models that rely on specific transgenic mice (e.g. MISTRG) come with limitations related to scalability, cost, and applicability across cancer types.

Cancer models are key tools for assessing efficacy of novel therapeutics prior clinical trials. In the era of personalized medicine, in depth characterization of the models at the omics levels is crucial to link treatment outcomes to specific tumor profiles [6, 15]. Nowadays, advanced models allow for detailed molecular analyses upon treatment in time and space, an assessment that cannot be performed in patients. Still, the advancements of preclinical cancer modeling come at a price. Preclinical testing requires more advanced readouts as the model becomes more complex. Biochemical assays commonly applied in classical cell cultures are not appropriate for complex models. High-throughput readouts based on imaging combined with novel analytical algorithms will be instrumental in discriminating drug efficacy in tumor cells and associated TME components.

While quoting a famous aphorism in statistics by George E. P. Box: ‘’All models are wrong, but some are useful”, we expect that development of a plethora of preclinical models will allow us to tailor cancer modeling towards specific and clinically-relevant studies. In recognition of the important field, we are now welcoming submissions to our new Collection of articles titled ‘Advances in pre-clinical cancer models’. More details can be found here: https://www.biomedcentral.com/collections/apcm. We hope that this Collection will provide a useful platform for novel protocols and discoveries advancing preclinical cancer modeling. We aim to discuss diverse modeling options in vitro, ex vivo and in vivo.

## Data Availability

Not applicable.
